# Author Correction: Multi-qubit Quantum Rabi Model and Multi-partite Entangled States in a Circuit QED System

**DOI:** 10.1038/s41598-019-46781-w

**Published:** 2019-07-17

**Authors:** Jialun Li, Gangcheng Wang, Ruoqi Xiao, Chunfang Sun, Chunfeng Wu, Kang Xue

**Affiliations:** 1 Center for Quantum Sciences and School of Physics, Northeast Normal University, Changchun, 130024 China; 2 Science and Mathematics, and Pillar of Engineering Product Development, Singapore University of Technology and Design, 8 Somapah Road, Singapore, 487372 Singapore

Correction to: *Scientific Reports* 10.1038/s41598-018-35751-3, published online 04 February 2019

This Article contains errors in the Reference section.

“5. Irish, E.-K. Erratum: Generalized Rotating-Wave Approximation for Arbitrarily Large Coupling. *Phys. Rev. Lett*. 99, (173601 (2007).”

should read:

“5. Irish, E.-K. Generalized Rotating-Wave Approximation for Arbitrarily Large Coupling. *Phys. Rev. Lett*. 99, 173601 (2007).”

In addition,

“68. Goerz, M., Motzoi, F., Whaley, K., Koch, C. & Nori, F. Charting the circuit QED design landscape using optimal control theory. *npj Quantum Information* **3**, 37 (2017).”

should read:

“68. Goerz, M., Motzoi, F., Whaley, K. & Koch, C. Charting the circuit QED design landscape using optimal control theory. *npj Quantum Information* **3**, 37 (2017).”

